# Sources of Nitrogen-, Sulfur-, and Phosphorus-Containing Feedstocks for Prebiotic Chemistry in the Planetary Environment

**DOI:** 10.3390/life12081268

**Published:** 2022-08-19

**Authors:** Zoe R. Todd

**Affiliations:** Department of Earth and Space Sciences, University of Washington, Seattle, WA 98195, USA; ztodd@uw.edu

**Keywords:** prebiotic chemistry, origins of life, feedstocks, planetary environments

## Abstract

Biochemistry on Earth makes use of the key elements carbon, hydrogen, oxygen, nitrogen, phosphorus, and sulfur (or CHONPS). Chemically accessible molecules containing these key elements would presumably have been necessary for prebiotic chemistry and the origins of life on Earth. For example, feedstock molecules including fixed nitrogen (e.g., ammonia, nitrite, nitrate), accessible forms of phosphorus (e.g., phosphate, phosphite, etc.), and sources of sulfur (e.g., sulfide, sulfite) may have been necessary for the origins of life, given the biochemistry seen in Earth life today. This review describes potential sources of nitrogen-, sulfur-, and phosphorus-containing molecules in the context of planetary environments. For the early Earth, such considerations may be able to aid in the understanding of our own origins. Additionally, as we learn more about potential environments on other planets (for example, with upcoming next-generation telescope observations or new missions to explore other bodies in our Solar System), evaluating potential sources for elements necessary for life (as we know it) can help constrain the potential habitability of these worlds.

## 1. Introduction

Life on Earth today is primarily made up of the elements carbon, hydrogen, oxygen, nitrogen, phosphorus, and sulfur, along with a number of minor elements. While many theories for the origins of life and the synthesis of prebiotically relevant compounds exist, and are a topic of active research, the origins of life presumably needed chemically active sources of these critical elements for prebiotic synthesis. For the origins of life to be possible—or even likely—to occur on a planet, the planetary environment would need to have readily available sources of these elements. With relevant feedstock sources present, prebiotic synthesis and, ultimately, the origins of life could potentially occur. 

Past work has suggested various syntheses of prebiotically relevant compounds, including, for example, amino acids [1], nucleobases [2,3], nucleotides [4,5,6,7], and sugars [8,9] from various types of feedstock molecules. While the precise path that the origins of life occurred on Earth is currently still a mystery (and may always be uncertain), investigating prebiotic synthesis considering the environmental conditions readily available on a planet can help constrain the possibilities for prebiotic chemistry. Even beyond investigating our own origins on Earth, understanding possible pathways leading to the origins of life under different planetary conditions can help to aid the search for life on other planets. 

This review presents the possible sources of various feedstock molecules providing the crucial elements for life on Earth, namely, nitrogen, sulfur, and phosphorus, with the goal of extending possible characterizations beyond what is thought to be relevant for the early Earth in order to provide some relevance to possible exoplanetary environments. In particular, this review addresses the possible sources and speciation of nitrogen-, sulfur-, and phosphorus-containing molecules that may be relevant feedstocks for prebiotic chemistry and the origins of life. In addition to considering sources of molecules containing these elements, the environments favoring the presence and possible accumulation of such molecules are considered, with the aim to offer some constraints on potential prebiotic synthesis under a range of conditions. 

As new and more powerful telescopic observations of exoplanets become available, and as missions seek to better characterize the environments on planets and moons within our own Solar System (and perhaps, ultimately, beyond), considerations of the presence of potential crucial feedstock molecules can inform potential environments for prebiotic chemistry to help understand the potential for habitability beyond our planet. 

## 2. Nitrogen

**Figure 1:**
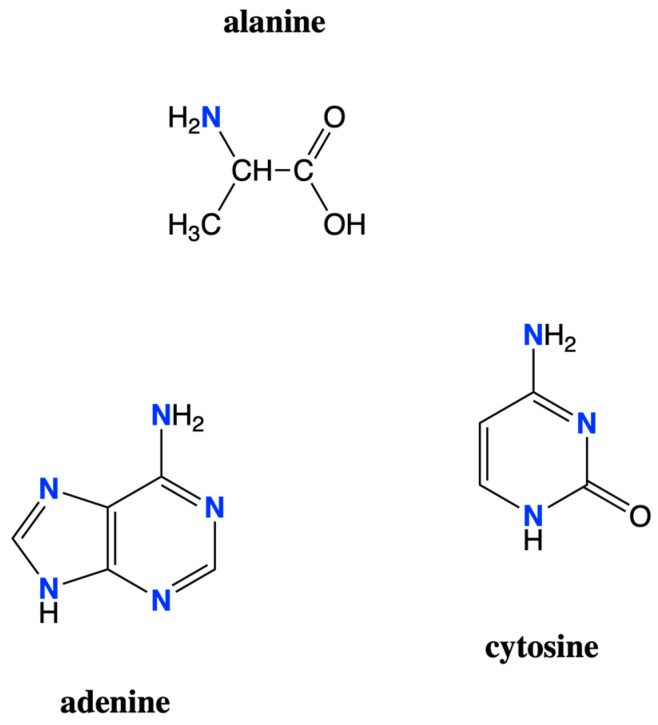
Chemical structures of example biomolecules containing nitrogen. Amino acids, such as alanine, contain nitrogen in their amine groups. The nitrogenous bases of DNA and RNA, such as the adenine and cytosine shown here, are also examples of biomolecules containing nitrogen.

Nitrogen plays a critical role in the biochemistry of Earth life, with roles as a component of amino acids, RNA and DNA, and a number of other important molecules for life. Amino acids are the building blocks of proteins, which have a number of important roles in life. Proteins act as enzymes to catalyze chemical reactions necessary for life and can also be hormones, neurotransmitters, structure molecules, etc. Nucleotides, the monomers of DNA and RNA, contain nitrogenous bases (A, G, C, T, and U), which are the letters used in life’s genetic information. Figure 1 shows examples of prominent biomolecules containing nitrogen.

Nitrogen is present in significant quantities on Earth, with our modern atmosphere being made of 78% dinitrogen (N_2_). However, despite the large abundance of nitrogen on Earth, this element can be the limiting nutrient for life today. This is due to the triple bond in N_2_ contained in the atmosphere; the high energy required to break apart N_2_ renders it largely inaccessible to life. To be useful for life (or pre-life), nitrogen must be “fixed” to more chemically accessible forms. The precise atmospheric content during the origins of life on Earth is not known, but generally, a dinitrogen-dominated atmosphere is favored, with a range of possible partial pressures (see Catling and Kasting [10] and references therein). Furthermore, nitrogen-containing compounds, such as N_2_O, could have been important greenhouse gases to combat the so-called Faint Young Sun Paradox [11]. 

**Figure 2:**
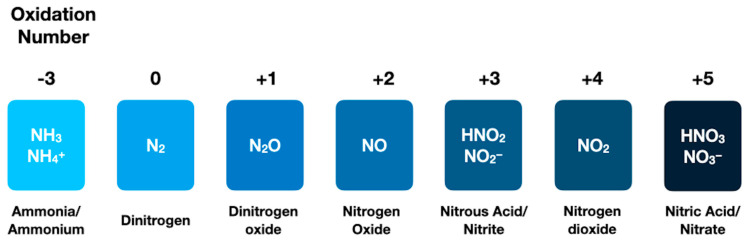
Nitrogen can range in oxidation number from −3 to +5, and, consequently, take on a number of different forms. These include, for example, ammonia/ammonium, dinitrogen, dinitrogen oxide, nitrogen oxide, nitrous acid/nitrite, nitrogen dioxide, and nitric acid/nitrate.

Nitrogen can range across a variety of oxidation states (−3 to +5), with possible forms including ammonia/ammonium (NH_3_/NH_4_^+^), dinitrogen (N_2_), nitrite (NO_2_^−^), or nitrate (NO_3_^−^), shown in Figure 2.

On modern Earth, nitrogen is fixed primarily by biological or anthropogenic sources. Nitrogen fixation by life is thought to have first come about ~3.5 Gyr ago [12]; prior to this event, nitrogen fixation must have occurred through abiotic processes, such as lightning, photochemistry, or volcanism, for example, in order to yield chemically accessible forms of nitrogen. Nitrogen-containing compounds are potentially available from a variety of sources, including exogenous delivery and endogenous production mechanisms on a planet. 

### 2.1. Exogenous Sources of Nitrogen

#### 2.1.1. Interstellar and Circumstellar Environments

Many nitrogen-containing species have been detected in the interstellar medium, dating back to the first detection of a polyatomic interstellar species, ammonia [13]. Nitrogen can be present as a gas, either in atomic or molecular forms in molecular clouds [14]. Atomic nitrogen has been detected in the diffuse ISM [15], while N_2_ has been detected in the far-UV toward a background star [16]. 

Ammonia has been detected in infrared dark clouds [17,18], molecular clouds [19,20], and the galactic center [21,22]. Nitrogen-containing compounds derived from ammonia, such as N_2_H^+^ and N_2_D^+^, have also been detected in environments with higher densities and used to probe their characteristics [23,24,25]. Nitriles, which contain a C-N triple bond, have been detected in both diffuse and dense interstellar environments [26,27,28]. 

In interstellar environments, nitrogen carriers are typically volatile, meaning that the incorporation of nitrogen into forming planets is not necessarily straightforward. Instead, a planet’s nitrogen may come from sources such as asteroids, comets, or N_2_ from the solar nebula [29,30,31,32,33,34,35]. Nitrogen may also be incorporated into dust grains [36] or molecular ices such as HCN and NH_3_; these may also serve as sources of nitrogen as planets are forming [37]. On average, ices located toward low-mass young stellar objects contain ~10% of the total nitrogen, with the remaining nitrogen expected to be gaseous N or N_2_ [38]. NH_3_ has been detected toward dense cores [39,40]; HCN has been detected in protoplanetary disks [41,42]. The irradiation of interstellar ice analogs has been shown to produce a variety of amino acids [43,44,45]. 

#### 2.1.2. Solar System

Nitrogen is found in many planets and bodies in the Solar System in addition to the Earth. Venus’s atmosphere contains a greater absolute quantity of nitrogen than that of the Earth [46]. The Martian atmosphere contains ~2% N_2_ [47,48], and oxidized nitrogen compounds have been detected in Martian sediments [49]. In addition to the rocky planets, icy outer bodies, including Titan, Triton, and Pluto, contain dinitrogen in their atmospheres [50]; some may also contain nitrogen ices (e.g., Pluto [51]). Indeed, Titan hosts a substantial atmosphere, first confirmed in 1944 [52], dominated by dinitrogen [53]. The atmospheric photochemistry on Titan can generate a variety of interesting organic species (see Willacy et al. [54] and references therein; Horst [55] and references therein). 

Comets contain a number of nitrogen-containing species, including HCN, HNC, HNCO, CH_3_CN, HC_3_N, and NH_2_CHO [56,57]. Furthermore, N_2_ has been detected for the first time in situ in comet 67P/Churyumov–Gerasimenko by ROSINA/Rosetta [58]. Comet 67P/Churyumov–Gerasimenko has been suggested to contain ammonium salts on the surface, due to a detected absorption band near 3.2 microns [59]. 

Many bodies in the Solar System show depleted values of nitrogen when compared to the Sun and the ISM. Distant comets, including Halley and Hale–Bopp, contain roughly less than one order of magnitude depletion in nitrogen, while meteorites are 1–3 orders of magnitude depleted in nitrogen, compared to the Sun [60]. The Earth is depleted by about five orders of magnitude in nitrogen, which could be explained by a significant amount of nitrogen present in the Earth’s interior [61,62]. 

Nitrogen has also been detected in various classes of meteorites [63]: CI, CM, and CR chondrites contain 500–2000 ppm total N in the form of soluble and insoluble organics. CO, CV, and CK meteorites contain 5–200 ppm total N, while ordinary and enstatite chondrites contain smaller quantities of total N. Iron meteorites contain 100–200 ppm N and Martian meteorites have 1–4 ppm nitrogen [63]. 

In addition, some nitrogen-containing heterocycles have been detected in meteorites [64]. Pyridine-monocarboxylic acids were detected in carbonaceous chondrites, including Tagish Lake and Murchison, with abundances around ~7 ppm [65,66,67,68]. This raises the intriguing possibility that the delivery of some RNA- or DNA-like heterocycles could have been possible on the early Earth. Diketopiperazine and hydantoins were also detected in carbonaceous chondrites, along with claims of purines, pyrimidines, triazines, pyridines, and quinolines [64]. 

Furthermore, meteorites have been shown to contain numerous identifiable amino acids and related compounds [69,70]. It has long been questioned if amino acids found in meteorites show enantiomeric excesses for the L-enantiomer that is used in life on Earth; see Glavin et al. [70] and references therein for further discussion. 

### 2.2. Endogenous Sources of Nitrogen

#### 2.2.1. Lightning

Lightning can be a potential abiotic source of fixed nitrogen on planets. The high amounts of energy produced during lightning events passing through a nitrogen-containing atmosphere can induce chemical reactions. A lightning bolt will create a shock wave of air with high temperatures in which chemical reactions between various gases in the atmosphere can occur and quickly reach equilibrium. The temperature of the shocked air decreases as it expands until, eventually, chemical reaction rates are too slow to reach chemical equilibrium. The temperature at which this occurs is the “freeze out” temperature [71]. 

The oxidation state of the atmosphere and the ratio of elements can contribute to the overall speciation of fixed nitrogen generated from lightning synthesis [71,72]. Chameides and Walker [72] found that the production rates of HCN and NO strongly depend on the C/O ratio, with HCN favored when C > O, and NO favored when C < O. Lightning in an N_2_/CO_2_-dominated atmosphere can produce significant quantities of NO, though the precise amounts depend on the ratio and amount of N_2_ and CO_2_ [71]. In atmospheres with lower abundances of CO_2_, the lightning-driven production of NO would have been lower [73].

Coronal discharges, occurring from pointed objects, can also fix nitrogen into NO_x_ species, including NO and N_2_O, though likely not in significant quantities on the early Earth compared to other sources of fixed nitrogen [74]. 

#### 2.2.2. Photochemistry

Another abiotic source of fixed nitrogen production in the atmosphere is due to photochemistry. For example, Zahnle [75] outlined a process where extreme ultraviolet radiation (λ<102 nm) from the Sun generates N atoms in the upper atmosphere. These N atoms then undergo further chemical reactions to form products such as HCN and NO, with varying amounts depending on the redox state of the atmosphere. More reducing atmospheres favor HCN production, while this mechanism is less efficient in more oxidizing atmospheres and tends to favor oxidized products such as NO. Again, the C/O ratio plays an important role in partitioning between species, with HCN favored when C/O > 1 [76]. 

Atmospheric N_2_ can be converted into ammonia (NH_3_) by using photochemistry and titanium oxide (TiO_2_) as a catalyst [77,78]. This process can occur in natural environments containing enhanced abundances of titanium, such as some deserts (e.g., Imperial Sand Dunes, California [79]). Henderson-Sellers and Schwartz [79] suggested this could have been a relevant source of NH_3_ on the early Earth; however, ammonia has a relatively short lifetime to UV photodegradation [80,81]. 

#### 2.2.3. Impact Production

During impacts, a high-energy body passes through a potentially dinitrogen-rich atmosphere, creating high temperatures and pressures that can invoke various chemical reactions, resulting in the production of fixed nitrogen species. For example, Fegley et al. [82] studied the formation of HCN as a result of impact shocks; they found this process to be efficient in atmospheres where the C/O ratio is >1. Parkos et al. [83] modeled the production of HCN from atmospheric heating due to impacts, using equilibrium chemistry and a correction factor to account for nonequilibrium reactions. At lower C/O ratios, NO production is generally favored over HCN [71,84]. Impacts can also yield high-temperature plasma in the post-impact plume, which could be the site of synthesis for nitrogen-containing molecules including amino acids and nucleobases [85,86]. 

#### 2.2.4. Volcanism

Volcanism is another potential abiotic source of fixed nitrogen. Atmospheric N_2_ can be fixed in hot volcanic vent environments to species including NO and NO_2_ [87]. The early Earth likely experienced some episodes of major volcanism, which could have generated significant amounts of fixed nitrogen [87,88]. 

Volcanic lightning is another phenomenon that could have produced fixed nitrogen on the early Earth. Volcanic eruptions can create plumes and clouds in which lightning occurs at a high frequency (~10–100 flash/min [89,90]). Given the possible high rates of volcanism on the early Earth, volcanic lightning may have contributed to the production of fixed nitrogen [91]; this process has also been suggested to be plausible on early Mars [92]. 

#### 2.2.5. Cosmic Rays and Solar Particles

Cosmic rays are high-energy particles (typically protons or other atomic nuclei) generated from our Sun, other stars, and even distant galaxies. These particles travel through space at high speeds and can deposit significant amounts of energy in the atmosphere when passing through. As a result, N_2_ in the atmosphere can be broken into N atoms, which can then undergo further chemical reactions to form fixed nitrogen species, such as NO [93] and other NO_x_ species [94]. Stars also emit high-energy particles, from stellar winds, coronal mass ejections, or stellar flares [95]; the prebiotic synthesis of amino acids and nucleobases is possible due to high-energy irradiation from both solar particles and cosmic rays [96]. 

#### 2.2.6. Mineral Reduction

Minerals have been shown to reduce nitrogen species, including N_2_, NO_2_^−^, and NO_3_^−^, to NH_3_ [97,98]. The conditions for these reactions include hot temperatures (300–800 °C) and higher pressures (0.1–0.4 GPa), which can occur in hydrothermal systems [97]. Iron sulfides are present in many hydrothermal systems; these minerals have been shown, experimentally, to convert nitrite and nitrate into ammonia at higher temperatures [97,98]. 

### 2.3. Nitrogen in the Planetary Environment

As discussed above, NO is a commonly formed species of fixed nitrogen produced in the atmosphere from a number of sources, including lightning, photochemistry, volcanism, etc. Further chemical reactions act to process NO synthesized in the atmosphere to other fixed nitrogen species, potentially of use for prebiotic chemistry. NO and NO^−^ can react with themselves or each other to form species including N_2_O_2_^−^ and N_3_O_3_^−^, which quickly decay into N_2_O, NO_2_^−^, and NO_3_^−^ (see [71] and references therein). Once present in aqueous environments, NO_x_ species, including nitrite and nitrate, can undergo further degradation, e.g., due to photochemistry, processing in hydrothermal vents, reactions with ferrous iron, etc., which could limit the concentrations of these species available for prebiotic chemistry [99]. 

However, some of these degradation reactions of NO_x_ species can generate reduced nitrogen, including NH_3_, which could be of use for prebiotic chemistry. Ferrous iron, which was likely abundant on the pre-oxygenated early Earth, reacts with nitrate and nitrite to form ammonia [100]. Such reactions are more favorable at basic pHs (>7.3) and warm temperatures (>25 °C) [100]. 

Nitrogen plays a major role in life’s biochemistry on Earth and is present throughout the cosmos and Solar System. As a major constituent of Earth’s atmosphere, nitrogen itself is seemingly abundant on rocky planets [46], but the chemically/biochemically useful forms of fixed nitrogen are less abundant. However, a number of abiotic sources of fixed nitrogen are possible on planets, including lightning, photochemistry, impacts, volcanism, cosmic rays, and mineral reduction. 

## 3. Sulfur

**Figure 3:**
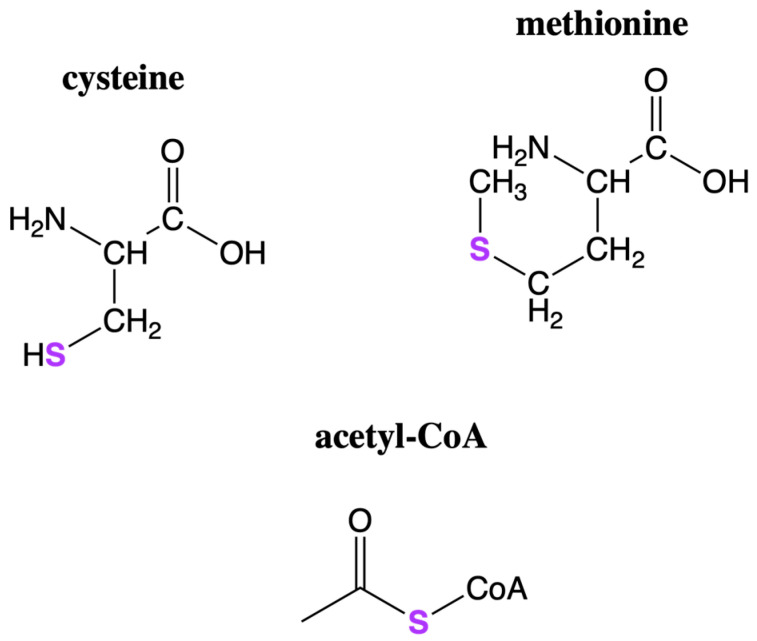
Chemical structures of example biomolecules containing sulfur. Two amino acids, cysteine and methionine, contain sulfur atoms. Sulfur is also found in coenzymes, such as acetyl-CoA, an important molecule in metabolism.

Sulfur is a crucial element of life due to its presence in a number of important biomolecules and coenzymes. Two of the twenty amino acids used by life today as components of proteins contain sulfur: methionine and cysteine. Methionine is a key amino acid, given that it initiates nearly all protein sequences in eukaryotes; the presence of sulfur in cysteine allows for the formation of disulfide bridges, which aid in protein structure and folding [101]. Sulfur also plays a role in many coenzymes, including acetyl CoA, a central player in metabolism [102], as well as in many cofactors through iron–sulfur clusters. Iron–sulfur clusters are used in electron transport to harness energy in life today; they are highly conserved across various taxa, indicating that their role in life’s biochemistry may be ancient in origin [103]. The chemical structures of some important sulfur-containing biomolecules are shown in Figure 3. 

Sulfur and sulfur-containing compounds have commonly been implicated in prebiotic chemistry across a wide range of possible syntheses and hypotheses. Wächtershäuser [104] proposed the “iron–sulfur world” hypothesis, where iron sulfide minerals play a key role in driving metabolic reactions used by the first life. Similarly, the “thioester world” was proposed by de Duve [105], where thioester molecules, potentially available on the early Earth, could provide the energy for driving other critical reactions for prebiotic chemistry.

Other hypotheses for the origins of life, where sulfur-containing molecules are not necessarily central, also implicate sulfur compounds as potentially important for prebiotic chemistry. For example, the cyanosulfidic chemistry proposed by the Sutherland Lab [106,107,108] makes use of sulfide or sulfite compounds, as do other prebiotic syntheses [5,109].

Sulfur is abundant on the Earth (10th most common element) and is present in the crust (0.03–0.1% abundance), the atmosphere, and the oceans [110]. Sulfur is present in its native form as well as in several sulfur minerals, including pyrite (FeS_2_) and gypsum (CaSO_4_) [111]. However, the crustal abundance of sulfur on Earth is depleted due to the partitioning of sulfur to sulfide magmas and the interior of the Earth [112]. Sulfur is volcanically released from Earth’s interior into the atmosphere in either the form of H_2_S or SO_2_ [111]. For details regarding the sulfur cycle on Earth, see Sievert et al. [113] and references therein. 

**Figure 4:**
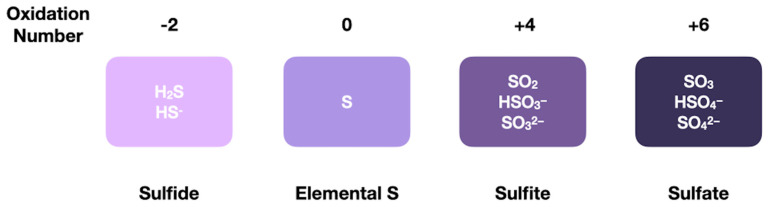
Sulfur can take on a number of chemical states, including hydrogen sulfide, elemental sulfur, sulfite, and sulfate. Sulfur spans oxidation numbers from −2 to +6.

Sulfur can take on various forms, including sulfides, elemental sulfur, sulfite, and sulfate, with oxidation states ranging from −2 to +6 (see Figure 4), in addition to various types of organosulfur compounds. On the early Earth, when oxygen was less abundant, more reduced forms of sulfur may have been favored [114]. On modern Earth, more oxidized forms of sulfur are favored in certain environments, such as the ocean [115]. 

Sulfur-containing compounds are available from a number of sources on planets, including both exogenous and endogenous. Exogenous sources of sulfur compounds are discussed first, before moving on to the endogenous sources of sulfur compounds. 

### 3.1. Exogenous Sources of Sulfur

#### 3.1.1. Interstellar and Circumstellar Environments

A number of sulfur-containing molecules have been detected in interstellar or circumstellar environments, including prestellar cores [116], protostellar envelopes [117], and photodissociation regions (PDRs) [118]. 

Interstellar CS was first detected in 1971 by Penzias [119] toward four sources, while OCS and SO_2_ were detected in molecular clouds [120,121]. Interstellar H_2_S has also been detected, e.g., toward galactic sources [122]. Ionized sulfur compounds, including HS^+^, have also been reported [123,124]. Organic thiols, such as CH_3_SH (methyl mercaptan), g-C_2_H_5_SH (ethyl mercaptan), the trans-isomer of monothioformic acid [125], and thioformaldehyde (H_2_CS) [126], as well as other sulfur-containing molecules, have also been detected. 

In the diffuse interstellar medium, sulfur is present near cosmic abundances; however, sulfur is generally depleted in abundance in environments such as molecular clouds and star-forming regions [127]. The number of sulfur-containing species currently detected accounts for only a small fraction of the total expected abundance [128,129], leading to the suggestion that there is a source of sulfur yet to be identified. 

The sulfur chemistry of protoplanetary disks is an area of continuing work; observations of species including CS and H_2_CS have been made in several protoplanetary disks [130], with the goal of understanding chemical inheritance from the ISM and the ultimate chemical composition of forming planets. 

#### 3.1.2. Solar System

Sulfur is found throughout our Solar System and, presumably, in other planetary systems as well. Mercury contains solid elemental sulfur in the polar regions [131], while sulfate minerals are found on Mars [132,133,134]. The Earth contains abundant amounts of sulfur, as described previously. Furthermore, sulfur species can be found in the atmospheres of planets, including H_2_S in the atmospheres of Uranus and Neptune [135,136] and atomic sulfur and sulfur monoxide in Io’s atmosphere [137,138] (Feaga et al., 2002, Lellouch et al., 1996). Venus’s atmosphere contains SO_2_ [139,140] and sulfuric acid clouds (see Dai et al. [141] and references therein). 

Sulfur and sulfur-containing species are also found in smaller bodies in the Solar System. Species including H_2_S, OCS, SO_2_, H_2_CS, and S_2_ have been detected in comets [56,142]. Carbonaceous chondrites contain ~5.41% sulfur on average [143]. Meteoritic samples contain various sulfur species, including the mineral troilite (FeS) in iron meteorites, while sulfides, elemental sulfur, and sulfates are found in stony meteorites [144]. 

Sulfur-containing species could potentially have been delivered to planets through impact events on larger bodies [145] or smaller bodies, such as those responsible for many meteorites found on Earth. Even if the intact delivery of molecules containing sulfur does not occur during an impact, significant amounts of sulfur can be introduced to the atmosphere during these events; this sulfur can then undergo further chemistry and photochemistry in the atmosphere to produce a range of species, including sulfuric acid [146]. 

Interplanetary dust particles (IDPs) can contain sulfur compounds, including sulfides [147,148], and could deliver such species during ablation in the planetary atmosphere [149]. 

### 3.2. Endogenous Sources of Sulfur

#### 3.2.1. Volcanism

The early Earth has been hypothesized to have experienced a higher level of volcanism than during modern times, perhaps due to a hotter interior and increased tectonic activity [150]. Volcanism outgassing can release sulfur-containing species, such as H_2_S and SO_2_, into the planetary atmosphere. The outgassing speciation of these compounds depends on the precise characteristics of the planet, including the redox state and the role of subvolcanic reactions [151]. Previous researchers have assumed a ratio of 10:1 SO_2_:H_2_S for the early Earth [152,153]. The outgassing rates for SO_2_ on the early Earth have been estimated as being roughly three times the modern outgassing rate [150,152,154], but higher rates are possible during major episodes of volcanism, such as those leading to the formation of the Deccan traps on Earth [155,156]. Once volcanically outgassed to the atmosphere, sulfur-containing species can react with minerals on the surface to form sulfur-containing mineral depositions. The isotopic fractionation of sulfur found in the geological record on Earth can offer clues as to the processes occurring on a planet (see [157] and references therein), including the Great Oxidation Event [158]. It is possible that the volcanic release of SO_2_ and subsequent photochemical reactions are responsible for the observed sulfur mass-dependent and mass-independent isotopic fractionation observed in ancient sediments [159,160]. 

The volcanic outgassing of SO_2_ and H_2_S into the planetary atmosphere can allow for the accumulation of sulfidic species in surface waters through Henry’s law of equilibrium. Ranjan et al. [161] calculated the expected concentrations of SO_2_- and H_2_S-derived sulfidic anions in surface waters on the early Earth as a function of parameters such as pH and sulfur outgassing rates. SO_2_, with a higher solubility and a more favorable acid dissociation constant (pKa) leads to higher overall available concentrations of SO_2_-derived anions, HSO_3_^−^ and SO_3_^2−^, than H_2_S-derived anions, HS^−^ and S^2−^ [161]. 

#### 3.2.2. Hydrothermal Vents

Hydrothermal vents are environments where chemically rich material from the interior of the Earth meets ocean water; the gradients in temperature, pH, and chemical concentration have been postulated to be potentially useful for driving prebiotic synthesis (e.g., Martin et al. [162] and references therein). There are two distinct categories of submarine hydrothermal vents: black smokers and white smokers; these differ in temperature and chemistry. Black smokers are hot (~350 °C) and are characterized by high concentrations of dissolved metals, sulfur compounds, and silica [163]. As the hot, chemically rich fluid from the vent interacts with the cooler surrounding seawater, metal sulfides, including Fe-, Cu, Zn-, and Pb-sulfides precipitate and generate the characteristic black color [163]. These metal sulfide precipitates form “chimneys” that can grow to large heights. The second type of hydrothermal vent, white smokers, are cooler (~50–90 °C) [162]. Fluids in white smokers are typically rich in calcium and commonly form sulfate and carbonate deposits [164]. An example is the Lost City hydrothermal system, which contains a large, ~60 m tall chimney [165]. Sulfur-containing molecules and minerals, including methanethiol (CH_3_SH) [166] and pyrite (FeS_2_) [167] have been detected in hydrothermal vents. It may be possible for such minerals to form nanoparticulates, which could potentially travel substantial distances from the vent [168]. Other metal sulfides, including Cu- and Zn-sulfides, are found in modern hydrothermal vent systems [169,170]. 

Metal sulfides are found in coenzymes used by life today and have been suggested to play an important role in the origins of life as proto-catalysts [171,172,173,174]. For example, Ni/Fe-S can catalyze the reaction of carbon monoxide (CO), hydrogen sulfide (H_2_S), and methyl mercaptan (CH_3_SH) to yield methyl thioacetate (CH_3_-CO-SCH_3_) [175]. For a more detailed discussion of the possibility of prebiotic chemistry occurring in hydrothermal settings, see [164,176] and references therein. 

## 4. Phosphorus

**Figure 5:**
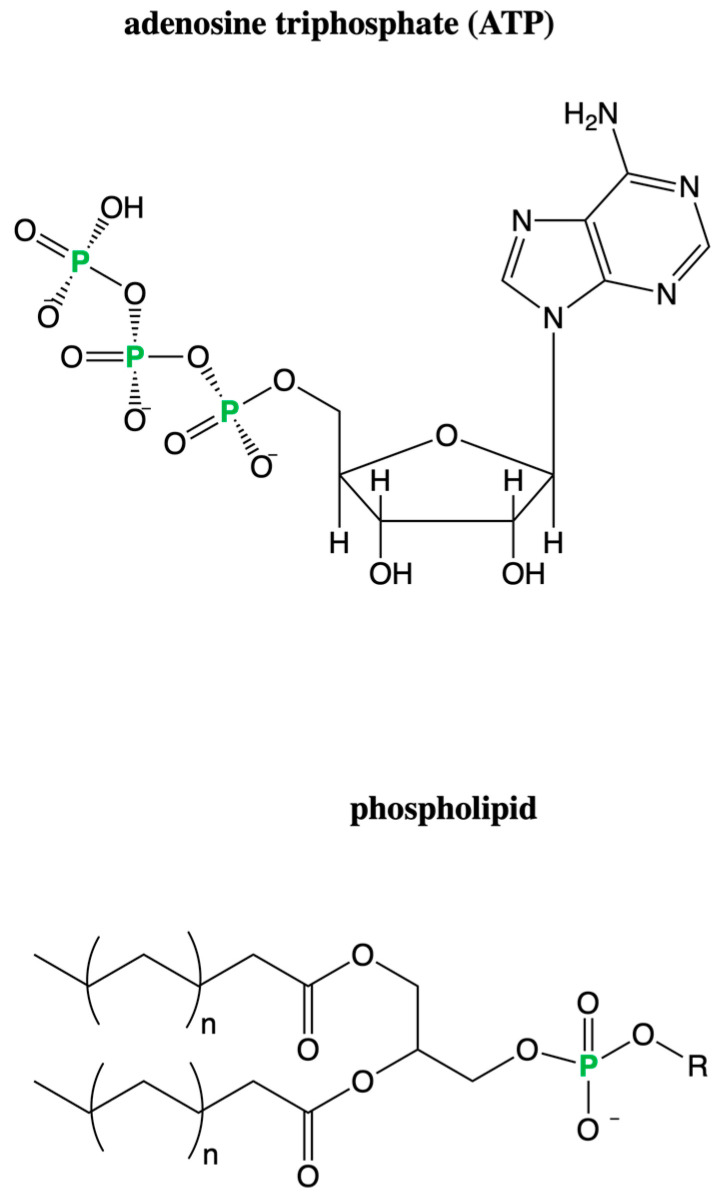
Chemical structures of example biomolecules containing phosphorus. Examples include adenosine triphosphate (ATP), which is the main energy currency used by life today, and phospholipids, which make up cell membranes.

Phosphorus plays an important role in biology, acting as a component of the phosphate backbone of nucleic acids and the phospholipids used to make up cell membranes. The phosphorylation of molecules is used for energy storage and use; for example, ATP hydrolysis provides cells with the energy to function. Figure 5 shows the chemical structures of several important phosphorus-containing biomolecules. Phosphorus is abundant in life, is present in large quantities in Earth’s crust (1.2 g P/kg, ranking 11th most abundant) [177], and has a relatively low solar abundance of 10^5.5^ atoms per 10^12^ H atoms [178]. 

**Figure 6:**
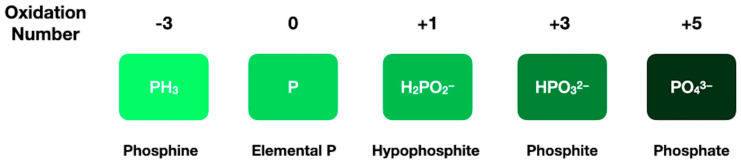
Phosphorus can range in its oxidation state from −3 to +5. Inorganic phosphate species include phosphine, elemental phosphorus, hypophosphite, phosphite, and phosphate.

The most common form of phosphorus used by modern biology is phosphate, PO_4_^3−^. However, phosphorus can exist in a number of forms, with oxidation states ranging from −3 to +5 (see Figure 6). Phosphorus can also be incorporated into organic molecules to form organophosphates. Typical forms of organophosphates include: (1) molecules containing a P-O-C bond where the carbon is sp^2^-hybridized, such as glycerate phosphate or phosphoenolpyruvate; (2) molecules with a P-O-P bond, e.g., those used for energy storage in life today, such as ATP; and (3) molecules containing a P-O-C bond, where the carbon is sp^3^ hybridized, such as phospholipids and nucleic acids [179]. 

The chemical form that phosphorus takes on a planet depends largely on the oxidation state of the environment. More reducing environments will favor the presence of reduced phosphorus species, including phosphine, hypophosphite, and phosphite. Phosphorus is unique from other elements important for life in that only one form is volatile: phosphine, and this form is disfavored under Earth’s conditions [179]. However, the most reduced form of phosphorus, phosphine, is present in the atmospheres of Jupiter and Saturn [180]. Earth’s current oxidation state thermodynamically favors orthophosphate as the dominant form of phosphate; more reducing forms are not thermodynamically stable under most conditions currently on Earth [181]. Orthophosphate is contained in minerals, such as apatite (Ca_5_(PO_4_)_3_(OH,F,Cl)) and olivine-type compounds ((Fe,Mg)_2_SiO_4_) [181,182,183,184,185]. The early Earth was likely more reducing, especially prior to the oxygenation of the atmosphere [186]. Earlier in the Earth’s history, other phosphorus-containing minerals likely included whitlockite (Ca_9_MgH(PO_4_)_7_) and brushite (CaHPO_4_·2H_2_O) [181]. 

Most minerals containing orthophosphates are relatively insoluble in water. For example, phosphate reacts with geochemically abundant ions such as Ca^2+^, Fe^2+^, Fe^3+^, and Al^3+^ to form apatite minerals and Fe- and Al-containing phosphates [187,188]. Consequently, the available concentrations of aqueous phosphate from equilibrium with orthophosphate-containing minerals are quite low. This has been a longstanding issue in prebiotic chemistry: How was phosphorus and/or phosphate available in sufficient concentrations to support proposed prebiotic chemical syntheses [182,189]? Various suggestions have been presented to address this issue; these will be discussed below. 

There are a number of potential sources of phosphorus and phosphorus-containing species on planets. Exogenous sources involve the delivery of phosphorus-containing compounds from extraterrestrial sources, while endogenous sources make use of phosphorus already contained on the Earth. 

### 4.1. Exogenous Sources of Phosphorus

#### 4.1.1. Interstellar and Circumstellar Environments

Phosphorus-containing species have been detected in a variety of astronomical environments, including in the interstellar medium (ISM) and circumstellar environments. The ISM contains a lower abundance of phosphorus than diffuse molecular clouds and star-forming regions; a plausible explanation for this observation is that phosphorus species can freeze out onto dust grains [190], thereby decreasing the observed abundance in the ISM. P^+^ has been observed in diffuse clouds [191] and toward stars [192,193]. Molecules containing phosphorus (e.g., PN, PO, HCP, CP, CCP, NCCP, PH_3_) have been detected toward circumstellar environments around C- and O-rich stars [194,195,196,197,198,199]. PO and PN have been detected in dense star-forming regions and molecular clouds in the Galactic Center [200,201,202,203,204,205,206]. However, the initial abundance of phosphorus in dense clouds is still uncertain [207].

#### 4.1.2. Solar System

In the Solar System, phosphorus is present in a variety of forms. During the formation of the Solar System, the condensation temperature of phosphorus (~1248 K) [208], determined the locations where phosphorus was present in volatile phases. At the location of Earth’s orbit, phosphorus may have been present in volatile forms during the process of planet formation [209,210]. In addition to volatile forms, phosphorus can reside in lithophilic (rock-loving) or siderophilic (metal-loving) forms, which selectively partition into the various phases of planetary bodies. Phosphorus present in a siderophilic phase will partition into the iron-rich core of a planetary body, which leaves less phosphorus available in the lithophilic form present in the bulk silicate phase of a planetary body. Indeed, the abundance of phosphorus in the Bulk Silicate Earth (BSE) is less than expected due to the loss of phosphorus from volatility alone [211,212]. During mantle melting, phosphorus can behave as an incompatible element, which increases its concentrations in mantle melts [210].

Phosphorus behaves similarly on other rocky planets in the Solar System. Lunar samples show the presence of the phosphate minerals apatite and whitlockite, as well as the phosphide schreibersite [213]. Phosphorus-containing compounds have been detected in Martian samples (e.g., from the Mars Exploration Rovers [214] and Mars Pathfinder [215]) and Martian meteorites (e.g., merrillite, a calcium phosphate mineral [216]). Indeed, the relative abundance of phosphorus on Mars appears to be greater than on Earth [213]. 

Many different types of meteorites contain phosphorus, including chondrites, achondrites, mesosiderites, and iron meteorites, with phosphorus on average being the 13th most abundant element found in meteorites [213,217]. The primary form of phosphorus depends on the meteorite type, with stony meteorites primarily containing phosphate minerals such as apatite-family minerals, whitlockite, and others [181]. Iron meteorites, pallasites, and enstatite chondrites primarily contain phosphorus in the form of phosphides, where phosphorus is in the P^3−^ form [181,210]. Schreibersite, (Fe,Ni)_3_P, is often implicated as a potential source of exogenous phosphorus and is of interest due to the resulting high availability of phosphorus [181]. When schreibersite is exposed to water, oxidation releases several phosphorus-containing species, including phosphite (HPO_3_^2−^), orthophosphate (HPO_4_^2−^), hypophosphate (HP_2_O_6_^3−^), and pyrophosphate (HP_2_O_7_^3−^) [218,219]. These species can further react to form other phosphorus-containing compounds under different conditions. For example, oxidizing conditions can yield peroxyphosphates (HPO_5_^2−^, HP_2_O_8_^3−^) [181]. Acidic conditions can yield phosphine (PH_3_), while UV irradiation provides hypophosphite (H_2_PO_2_^−^) [181]. However, the majority of phosphorus liberated from schreibersite oxidation is phosphite, HPO_3_^2−^ (>50% yield) [218,219]. Thus, schreibersite offers the advantage of yielding many various forms of phosphorus with relatively high solubility. 

Comets also contain phosphorus; in particular, the Rosetta mission to Comet 67P/Churyumov–Gerasimenko detected phosphorus [142], which was later suggested to be in the form of PO [220]. Interplanetary dust particles (IDPs), including those from the coma of Comet 67P/Churyumov–Gerasimenko, contain phosphorus [221]. In general, IDPs can contain both phosphates and phosphides [222], though Flynn et al. [223] found that oxidized phosphorus is favored. When entering the planetary atmosphere, interplanetary dust particles can undergo ablation and lose mass; the processing of vaporized P in the atmosphere could be a source of reduced phosphorus compounds [224]. The effect of ablation in liberating phosphorus and the subsequent phosphorus chemistry was investigated by Plane et al. [225], who found phosphorus can be delivered in the form of meteoric smoke particles and that the speciation of phosphorus after ablation depends on the oxygen fugacity of the atmosphere. From this source, phosphorus mainly takes the form of PO_2_ in the upper planetary atmosphere, which can then undergo further reactions to form PO and other species [226]. 

### 4.2. Endogenous Sources of Phosphorus

Phosphorus as an element is quite abundant on Earth; however, much of this phosphorus is locked up in fairly inaccessible mineral phosphate forms. Thus, the source of phosphorus itself on a planet is not necessarily in question; rather, the relevant issue is how to convert that phosphorus into more chemically usable forms. Reduced phosphorus compounds, such as schreibersite, phosphite, etc., are generally more reactive and accessible for potential prebiotic chemistry. Therefore, here, I outline a few mechanisms by which endogenous phosphate compounds on a planet could be converted to reduced phosphorus forms.

#### 4.2.1. Impact Production

Schreibersite, a potential source of phosphorus that has received much attention in the past, could be synthesized in situ on a planet in addition to being delivered exogenously. For example, the reduction of phosphate minerals can yield reduced phosphorus compounds, including schreibersite. Impactors containing significant amounts of metallic iron could reduce significant quantities of planetary material in the atmosphere and the vapor plume, which could potentially convert some phosphate-containing minerals into reduced phosphorus compounds, which are significantly more soluble. Lunar impact melts contain a majority of phosphorus in the form of schreibersite [227], which shows the plausibility of the delivery and/or formation of reduced phosphorus compounds [228]. 

#### 4.2.2. Hydrothermal-and Lightning-Driven Reduction

Furthermore, phosphate reduction can occur at low temperatures (e.g., <300 °C) [229], potentially in environments such as hydrothermal systems [230]. The presence of phosphorus in hydrothermal vents is suggested by the presence of nanometer-sized apatite crystals in several 3.46–2.46 Ga-banded iron formations (BIFs) and cherts in South Africa and Western Australia [231]. To convert phosphates to phosphides, highly reducing conditions are required, bringing the potential plausibility on the early Earth into question [229]. However, given the range of environments possible on exoplanets, perhaps there are scenarios in which hydrothermally derived reduced phosphorus compounds, including schreibersite, are possible. 

Alternatively, the reduction of phosphates at high temperatures (e.g., 500–1500 °C) could be another source of reduced phosphorus compounds [229]. This process may have been responsible for the detected natural schreibersite in the Haturium Formation in the Middle East [232], where nickel-rich rocks were exposed to high temperatures. 

Similarly, phosphide minerals are found in fulgurites [233,234], which are formed from the high temperatures released by cloud-to-ground lightning. Phosphate’s reduction to phosphides by lightning is generally incomplete [235] and only occurs under certain conditions; nevertheless, this could have been an additional source of reduced phosphorus on the early Earth [229,236].

### 4.3. Phosphorus in the Planetary Environment

While significant attention has been paid to reduced phosphorus compounds, due to their comparatively easier accessibility for prebiotic chemistry, it is also possible that some planetary environments allowed for the accumulation of significant concentrations of more oxidized phosphorus forms. Phosphate has traditionally been difficult to imagine in high concentrations on the early Earth due to its poor solubility in the presence of ions such as calcium, where the formation of apatite minerals is favored. One potential solution to this so-called “phosphate problem” is invoking the presence of carbonate lakes [237]. In carbonate-rich environments, calcium is sequestered by carbonates, allowing for the potential accumulation and concentration of aqueous phosphate, up to >1 M during evaporation [237]. Thus, the detailed geochemistry of local environments can influence the speciation, concentrations, and accumulation of phosphate. 

Phosphite, one of the favored products from dissolution and subsequent aqueous chemistry of schreibersite, can be oxidized to form phosphate in the presence of UV light and H_2_S/HS^−^ [238], yielding a potentially prebiotically plausible mechanism to form phosphates from meteoritically derived phosphite. Coupled with findings showing that the accumulation of phosphate may be possible in certain geochemical environments, perhaps this opens the possibility for oxidized phosphorus chemistry to be relevant to the origins of life, in addition to the traditional reduced phosphorus chemistry.

Given that phosphite is a major product of impact-derived schreibersite, Pasek [181] suggested that early Earth phosphorus geochemistry may have been primarily driven by phosphite. An advantage of phosphite over phosphate is increased solubility (~1000×) under similar pH and temperature conditions. Furthermore, phosphite is more reactive than orthophosphate, yielding condensed phosphates and organics containing C-P and C-O-P bonds [239]. Without a source of strong oxidizing agents, phosphite is stable, and it could potentially last on the early Earth for billions of years [181]. 

Phosphine (PH_3_), the most reduced form of phosphorus, has recently been the source of significant attention due to its potential detection in the clouds of Venus [240]. The abiotic production of phosphine may be possible on rocky planets from the ablation of large impactors near a thick cloud layer (such as the Venusian clouds) or the presence of reduced phosphorus compounds in a subcloud layer [241]. 

Overall, phosphorus plays a crucial role in Earth life today as a part of nucleic acids, phospholipids, and energy-carrying molecules such as ATP. Phosphorus is present cosmically, in environments including the interstellar medium and star-forming regions. Many planets and bodies in our Solar System contain significant quantities of phosphorus, in a variety of states. More oxidized forms of phosphorus are generally less accessible due to their minerals having lower solubilities; furthermore, these species tend to be less reactive than more reduced forms of phosphorus. Much attention has been placed on the phosphide mineral schreibersite, which can be delivered exogenously or produced endogenously on a planet. Schreibersite leads to a range of phosphorus-containing compounds when in an aqueous environment, with phosphite being the favored form. Phosphite may be relevant for prebiotic chemistry, or this species could be oxidized to the biologically used phosphate form. Polyphosphates, which are also potentially relevant for prebiotic chemistry, can be formed volcanically [242]. Lastly, the accumulation of significant concentrations of phosphate in aqueous solutions may not be as untenable as once thought when accounting for local geochemical environments, such as closed-basin alkaline lakes. Overall, there are many potential sources of phosphorus potentially available on planets for use in prebiotic chemistry. Indeed, the variety of relevant environments could suggest the possibility of significantly different phosphorus cycles on planets with varying conditions. 

## 5. Brief Discussion of Organics

The sources of organic species (i.e., potential carriers of the other most abundant elements used by life today, C, H, O) available on planets are briefly discussed here, but they are not described in detail, as this could be an entire review in and of itself. Suggested sources for further readings are presented below. 

### 5.1. Exogenous Sources of Organics

Organic species are present in the interstellar medium; protoplanetary disks; interplanetary dust particles; and primitive bodies including asteroids, comets, and interplanetary dust particles (see [56,243,244,245,246] and references therein). Organic delivery from exogenous sources has been suggested in the past (see [247,248,249] and references therein) as potentially important in seeding planets with the ingredients for life. The intact delivery of molecules, such as amino acids [250], and smaller feedstocks, such as hydrogen cyanide (HCN) [251], has been postulated. The chemistry occurring in post-impact vapor plumes can also lead to the formation of a variety of organic species (see [83,247,252] and references therein). 

### 5.2. Endogenous Sources of Organics

Atmospheric photochemical reactions can produce a variety of organic compounds; the types and amounts often depend on the composition of the primitive atmosphere and the redox state (see [247,253,254] and references therein). Volcanism is another potential source of abiotic organics (see [255,256,257] and references therein).

Serpentinization, a process that occurs to transform mineral species such as olivine and pyroxenes into other minerals, including serpentine, concomitantly results in the production of reducing species such as H_2_ and CH_4_ (see [258] and references therein). More complex organic synthesis, e.g., Fischer–Tropsch reactions that can form longer hydrocarbons [259,260], may be possible from these serpentinization reactions. 

The classical Miller–Urey spark-discharge experiment [1] showed that organics can be synthesized with simulated lightning in planetary atmospheres. The original Miller–Urey experiments used highly reducing atmospheres that may not have been present on the early Earth, but, nevertheless, they could be relevant for some exoplanets. Follow-up experiments have identified additional organic compounds (see [261,262] and references therein). For a deeper discussion of the influence of atmospheric composition on prebiotic synthesis from lightning, see Bada [263] and references therein. Chyba and Sagan [247] estimated the relevance of lightning-generated synthesis for the early Earth compared to other sources of organics. 

In summary, there are a wide variety of potential sources of organics on planets, including both exogenous delivery and endogenous production. Exogenous delivery can occur from, e.g., cometary impacts, meteorites, or interplanetary dust particles. Production on a planet can include mechanisms such as post-impact synthesis, atmospheric photochemistry, volcanism, lightning generation, serpentinization, and others. While the discussion provided here on organics is not comprehensive, the reader hopefully has the appropriate references for further details, if desired. 

## 6. Conclusions

Once a planet is seeded with the necessary elements, molecules, and feedstocks, prebiotic chemistry reactions may begin to proceed to build up chemical complexity and, perhaps, the precursors of life. There are numerous theories and mechanisms that have been outlined for the synthesis of precursors to life, including amino acids, fatty acids, nucleobases/sides/tides, and sugars. This review does not attempt to summarize these processes and hypotheses, as this could be the topic of a separate review, but rather stresses that the availability of feedstock molecules containing C, H, O, N, P, and S is just the beginning for the potential emergence of life. Furthermore, it is possible that, on other planets, a hypothetical emergence of life could use different elements and biochemistries than those present in Earth life. 

This review has outlined potential sources of nitrogen-, phosphorus-, and sulfur-containing feedstocks potentially available in planetary environments. In general, both exogenous (e.g., interstellar, cometary, meteoritic, IDP, etc.) and endogenous (volcanism, atmospheric chemistry, lightning generation, mineral geochemistry, etc.) are possible sources of these feedstock molecules (see Table 1 for a summary of sources discussed in this review). Depending on the characteristics of a planet, certain environments may prove to be more favorable for the production of the potentially important molecular carriers of these crucial elements for Earth life.

The aim of this review is to summarize potential sources and environments for nitrogen-, sulfur-, and phosphorus-containing feedstock molecules to aid in potentially constraining the origins of life on Earth and the possibility of the emergence of life on other planets.

## Figures and Tables

**Table 1:** Summary of potential sources of nitrogen-, sulfur-, and phosphorus-containing molecules in planetary environments discussed in this review.

Sources	Nitrogen	Phosphorus	Sulfur
*Exogenous*			
Interstellar/Circumstellar	✓	✓	✓
Solar System (planets, comets, meteorites, etc.)	✓	✓	✓
*Endogenous*			
Volcanism	✓	✓	✓
Hydrothermal	✓	✓	✓
Lightning	✓	✓	
Impact Production	✓	✓	
Photochemistry	✓	✓	
Cosmic Rays	✓		

## Data Availability

Not applicable.
